# Role of Lipids in Food Flavor Generation

**DOI:** 10.3390/molecules27155014

**Published:** 2022-08-06

**Authors:** Fereidoon Shahidi, Abul Hossain

**Affiliations:** Department of Biochemistry, Memorial University of Newfoundland, St. John’s, NL A1C 5S7, Canada

**Keywords:** flavor chemistry, lipid oxidation, Maillard reaction, volatile formation

## Abstract

Lipids in food are a source of essential fatty acids and also play a crucial role in flavor and off-flavor development. Lipids contribute to food flavor generation due to their degradation to volatile compounds during food processing, heating/cooking, and storage and/or interactions with other constituents developed from the Maillard reaction and Strecker degradation, among others. The degradation of lipids mainly occurs via autoxidation, photooxidation, and enzymatic oxidation, which produce a myriad of volatile compounds. The oxidation of unsaturated fatty acids generates hydroperoxides that then further break down to odor-active volatile secondary lipid oxidation products including aldehydes, alcohols, and ketones. In this contribution, a summary of the most relevant and recent findings on the production of volatile compounds from lipid degradation and Maillard reactions and their interaction has been compiled and discussed. In particular, the effects of processing such as cooking, drying, and fermentation as well as the storage of lipid-based foods on flavor generation are briefly discussed.

## 1. Introduction

Lipids, in addition to providing energy, texture, and mouthfeel, play an important role in the odor and flavor development of food. This could be due to the ability of lipids to generate odors and flavors, act as precursors of odor and flavor compounds, or modify the odor and flavor of other components [1]. Lipids are responsible for both the undesirable and desirable flavors of food; the oxidation of lipids mainly results in the development of off-flavor and lipoxygenase-derived lipid-based volatiles that are responsible for flavor generation. Food flavor is the manifestation of interactions between aroma, taste, and oral sensations, where the aroma is linked with mostly volatile compounds, and taste is associated with non-volatile high-molecular-weight components [2]. Generally, raw foods, mainly meat, provide little aroma and have a mild taste, but this can change to a strong aroma and taste upon processing. For example, odorants of raw and cooked sheep meat have been reported to be due to *cis*-1,5-octadien-3-one (geranium-like), 4-ethyloctanoic acid (mutton-like), *trans*-4,5-epoxy-2-decenal (metallic), and *trans*-2,4-decadienal (deep-fried). Moreover, flavor dilution (FD) factors of aroma compounds such as 2-acetyl-1-pyrroline, 4-hydroxy-2,5-dimethyl-3(2*H*)-furanone, and 2-aminoacetophenone were clearly increased upon cooking [3]. However, aquatic foods are slightly different as they have a comparatively strong flavor in their raw state. This is because of the presence of amines, lipoxygenase-derived lipid-based volatile components, and the development of secondary oxidation products in the raw stage [4,5]. On the other hand, lipids with low and high volatility are almost odorless and tasteless, respectively, partly because of their non-polar nature. 

Due to the lipid oxidation in food, an off-flavor is developed, and some bioactive compounds and fat-soluble vitamins are lost. The oxidation of unsaturated fatty acids is a complex process that can occur in the presence of oxygen or via non-enzymatic (autoxidation and photooxidation) and enzymatic (lipoxygenase) pathways. Lipid oxidation and the development of off-flavor compounds are catalyzed by light, heat, oxygen, photosensitizers, and transition metal ions (e.g., Fe^2+^ and Cu^2+^). Autoxidation occurs in the presence of triplet oxygen (^3^O_2_), while photooxidation happens in the presence of singlet oxygen (^1^O_2_) [6]. In autoxidation, primary oxidation products (e.g., hydroperoxides) are formed, and their subsequent break down into volatile secondary lipid oxidation products such as ketones, alcohols, and aldehydes, among others, are believed to play the main role in the development of an off-flavor. Due to the multiple methylene-interrupted *cis*-double bonds, polyunsaturated fatty acids (PUFAs) are highly prone to oxidation. For example, edible oils and fish lipids are rich in omega-3 fatty acids, where propanal and acrolein act as good indicators for assessing the degree of oxidation. Similarly, meat and meat products are rich in omega-6 fatty acids, and hexanal serves as a reliable indicator for flavor deterioration [7,8]. Moreover, heterocyclic compounds (e.g., pyrazines, oxazoles, pyridines, and thiazole) are formed mainly via non-enzymatic browning reactions, which generally play a key and desirable role in flavor generation [9]. On the other hand, upon thermal processing and heating experienced during frying, cooking, and grilling, hundreds of compounds can possibly be generated from lipid degradation, Maillard reactions, or Strecker degradation, which contribute to the overall desirable aroma upon interacting with each other. This could occur via forming a wide range of new volatile products upon interaction or entirely or partially blocking the presence of volatiles from others [10]. For instance, at both the initial and later stages of thermal processing, aldehydes that are produced upon the thermal degradation of lipids could join in the Maillard reactions [11]. In addition, the thermal generation of the oxidation products of lipids may be accompanied by the production of polar lipids and polymers that have no real effect on flavor [12]. Hence, the aim of this review is to summarize the most recent literature and knowledge of the role of lipids on flavor formation and their physiology and chemistry. An overview of the flavor generation of foods rich in PUFAs is also provided.

## 2. Physiology of Flavor

Flavor comprises taste, odor, aroma, and other mouth/oral cavity responses to texture, which is linked to the overall olfactory response to the food taken. Hence, flavor develops as a complex response of the mouth, tongue, nasal, and sinus cavities. Basic aroma, taste, and somatosensory perception are sensed by the olfactory bulb, gustatory sensory cells, and by trigeminal nerves, respectively [10]. Aroma, odor, and smell are synonymous terms used to describe olfactory responses, though aroma mostly refers to good flavor, while odor and smell are used to describe the off-flavor. Moreover, the term taste is related to the tongue response to sour, sweet, bitter, salty, and umami (monosodium glutamate (MSG)-like taste stimulated by MSG) sensations. Furthermore, astringent and metallic senses in the nasal and oral cavities can play an important role in flavor. Taste is mainly related to the high molecular weight of non-volatile lactic acid, quinine, sucrose, sodium chloride, and tannins, while the aroma is a sensual response when volatiles hit the back of the nose [2,13]. The interaction with the food matrix and static and dynamic factors is linked to the release of volatiles from food during chewing. For example, mass transfer, which is an example of a dynamic factor, mainly depends on the product viscosity and its interfaces; thus, volatiles can release more slowly in a viscous medium. Therefore, lipids act as modulators and transporters of volatiles in food [2].

The minimum concentration required to detect volatiles is known as the aroma threshold, which is mainly measured in parts per billion (ppb) or parts per million (ppm). For example, the thresholds for saturated, monounsaturated, and diunsaturated aldehydes are 0.014–1, 0.04–2.5, and 0.002–0.6 ppm, respectively, while the thresholds for alcohol, furans, and ketones are 0.001–3, 1–27, and 0.0002–5.5, respectively [6]. The polarity of the flavor compounds plays an important role in the flavor development. Usually, the odor and flavor potential is less in an oily medium than in an aqueous solution. Hence, low polarity fatty acids, mainly long-chain fatty acids, have a higher flavor threshold in an oily medium and a lower threshold in aqueous solutions. In contrast, polar compounds such as short-chain fatty acids have lower and higher thresholds in oils and water, respectively [1]. However, Maillard reaction-derived heterocyclic compounds containing sulfur and nitrogen have lower thresholds than those for lipid-derived volatiles. Therefore, greater/higher concentrations of lipid-derived components are required to exhibit the presence of aroma [10].

## 3. Role of Lipids Oxidation in Flavor or Off-Flavor Development

Lipids are susceptible to oxidation, which is believed to play the main non-microbial role in the quality degradation of food and food products including meat, fish, and oil-based products. Usually, off-flavors are linked very closely to lipids compared to proteins and carbohydrates. Lipid oxidation not only lessens the nutritional benefits of products due to the changes in essential fatty acids and vitamins but also negatively affects the sensory qualities such as flavor, texture, and color, which impacts on the overall consumer acceptance [6,14]. However, in some cases, lipid oxidation promotes the development of pleasant aromas, mainly during the ripening or dry-cured stages of meat products, which is one of the quality parameters of these products [15]. Lipid oxidation is a very complex phenomenon that undergoes a variety of reactions and produces a wide range of compounds. In a nutshell, fatty acids, mainly PUFAs, react with molecular oxygen and produce primary oxidation products (hydroperoxides) via free radical mechanisms. These compounds are highly unstable due to a weak oxygen–oxygen bond and are believed to make no contribution to odor and aroma development, but they break down rapidly and produce a wide variety of secondary constituents, which are responsible for the formation of off-flavors [16]. However, not all of these constituents similarly contribute to the flavor profiles, since the overall aroma perception is mainly dependent on the olfactory threshold, concentration, and type of products. Among these compounds, aldehydes seem to be the major compounds contributing to flavor development due to their low odor threshold and higher concentration. The most common aldehydes generated from lipid oxidation are *n*-alkanals, 4-hydroxy-2-alkenals, 2-alkenals, and malondialdehyde (MDA) [15]. Hence, lipid oxidation can occur in various pathways including autoxidation, photooxidation, thermal oxidation, and enzyme-catalyzed oxidation. Autoxidation is a very common lipid oxidation pathway, occurring through a continuous free-radical chain reaction. However, hydroperoxides are formed during the initiation stage of autoxidation, which is the main difference between the mechanisms of photo and enzymatic oxidation [12].

### 3.1. Formation of Oxidation Products via Autoxidation

**Figure 1:**
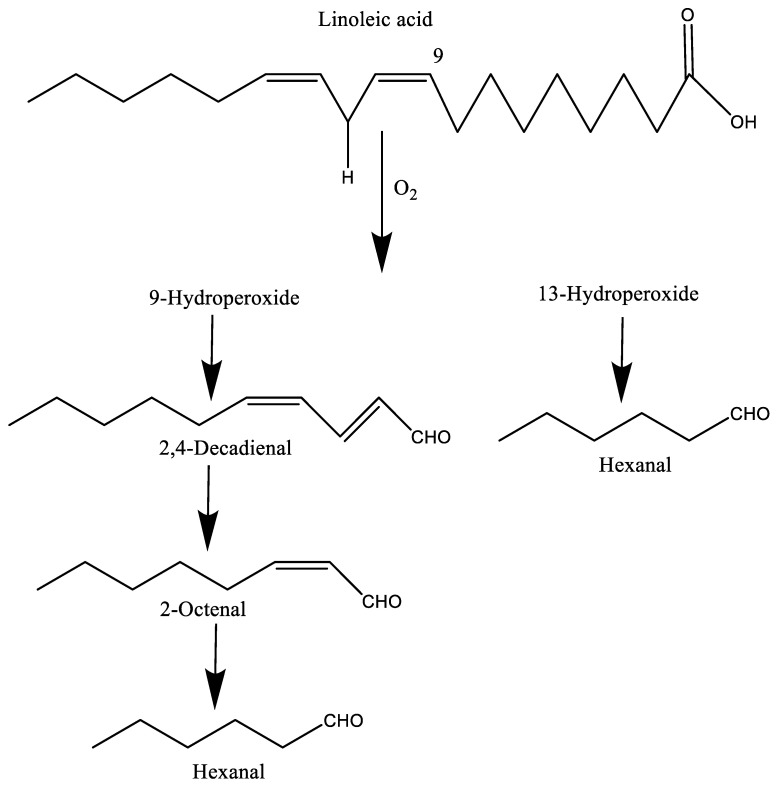
The formation of some volatiles from the autoxidation of linoleic acid.

Autoxidation is a combination of three different phases, namely, initiation, propagation, and termination. These steps are mainly responsible for producing radicals, followed by the multiplication of reactive compounds, finally degrading or reacting with each other in order to produce non-reactive products [17]. Autoxidation is the spontaneous oxidation of lipids in the presence of oxygen, mainly ^3^O_2_, which can interact with radical species due to its diradical nature. However, the interaction between unsaturated fatty acids (singlet electronic state) and oxygens (triplet electronic state) is not possible because of their chemical natures. Moreover, the triplet oxygen (^3^O_2_) cannot change its state to singlet states (^1^O_2_) without external support. Therefore, an initiator is necessary to convert ^3^O_2_ into ^1^O_2_, or reactive oxygen species (ROS) such as hydroxyl radicals, hydrogen peroxide (H_2_O_2_), superoxide anions, or to generate a radical by removing an electron from the unsaturated lipids [6,15]. The activation of oxygen mainly occurs in the presence of light, temperature, or metal ions such as Fe^2+^ and Cu^2+^. At the end of the propagation stage, primary oxidation products such as lipid hydroperoxides and conjugated dienes/trienes are produced and these further break down into a series of secondary oxidation products such as aldehydes, ketones, alcohols, hydrocarbons, epoxy compounds, and volatile organic acids. Among them, some compounds are responsible for the off-flavor at very low threshold values [12]. For example, linoleic acid produces 9- and 13-hydroperoxides in the presence of oxygen, and finally generates various volatiles including 2,4-decadienal, 2-octenal, and hexanal, among others, due to the cleavage of the C–O bond by alkoxy radicals derived from these hydroperoxides (Figure 1). Similarly, oleic acid forms 8-, 9-, 10-, and 11-hydroperoxides, which leads to many secondary compounds such as pentanal, octanal, hexanal, and heptanal, among others [9,13].

### 3.2. Formation of Oxidation Products via Photooxidation

A possible route for lipid-derived flavor generation is lipolysis, which raises the level of free fatty acids (FFAs). The FFAs may then be oxidized via various oxidation mechanisms and produces hydroperoxides, and further decompose to a series of volatile flavor compounds [18]. Photooxidation is much faster than autoxidation, occurring in the presence of singlet oxygen (^1^O_2_), which can be generated by ultraviolet (UV) light, and/or photosensitizers including riboflavin, chlorophyll, and myoglobin. Singlet oxygen is more reactive than triplet oxygen due to its higher electrophilicity. For instance, the interaction between ^1^O_2_ and linoleic acid is about 1500 times faster than that with ^3^O_2_ [19]. The photooxidation also produces hydroperoxides in many ways, where excited triplet sensitizers (e.g., myoglobin and hemoglobin) react with molecular oxygen (^3^O_2_) and produce ^1^O_2_. Subsequently, hydroperoxides are formed upon interactions between ^1^O_2_ and double bonds of unsaturated fatty acids without forming alkyl radical [20]. Moreover, ROS such as superoxide radical anion can be formed when the sensitizer interacts with ^3^O_2_, resulting in lipid oxidation due to the abstraction of hydrogen atoms from unsaturated fatty acids. In addition, hydroxyl radicals and ^1^O_2_ produced from the interaction between superoxide radical anions and H_2_O_2_ can initiate lipid oxidation in the presence of metal ions. Furthermore, alkyl radicals produced from the reaction of fatty acids and sensitizers can react with molecular oxygen and produce peroxyl radicals, initiating oxidation via a free radical chain mechanism [15]. Finally, primary oxidation products such as various hydroperoxides including 9- and 10-hydroperoxides for oleic acid, 9-, 10-, 12-, 13-, 15-, and 16-hydroperoxides for linolenic acid, and 9-, 10-, 12-, and 13-hydroperoxides for linoleic acid are formed, which lead to a series of volatile compounds [6]. The formation of these hydroperoxides mainly depends on the nature of the fatty acids involved in the oxidation reaction.

### 3.3. Formation of Oxidation Products via Enzymatic Oxidation

**Figure 2:**
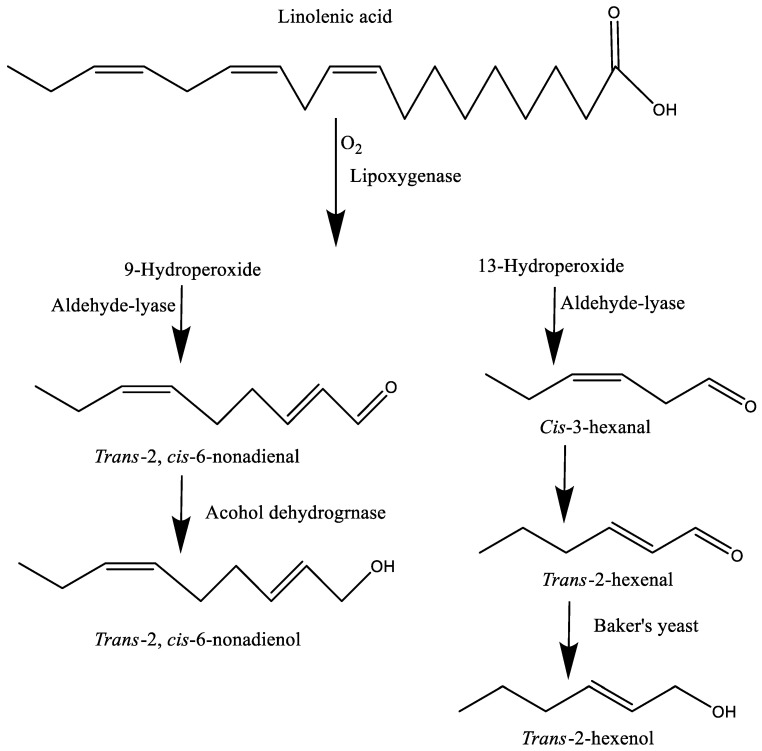
The formation of carbonyls and alcohols from linolenic acid.

Lipoxygenase, the main enzyme involved in enzymatic oxidation, is found abundantly in various species of plants, animals, and fish. It is a globular protein comprising a single polypeptide chain with a molecular mass of 75–80 kDa in animals and 94–104 kDa in plants. Lipoxygenase plays an important role in the degradation of unsaturated fatty acids, resulting in flavor generation. Lipoxygenase-mediated reactions produce the aromas of freshly harvested fish through the production of both volatile alcohols and carbonyl compounds [21]. For instance, lipoxygenase in fish can react with fatty acids and produces *cis*-4-heptenal and 2,4,7-decatrienal isomers, which are responsible for a fishy aroma [16]. The rate of enzyme-catalyzed lipid oxidation mainly depends on the concentration of enzymes, which is proportional to the oxidation [15]. Lipoxygenase produces conjugated hydroperoxides through oxidation when the active site of the enzyme abstracts a hydrogen atom from the methylene group of fatty acids. In order to exhibit activity, the active site of the enzyme containing iron needs to be in the ferrous form [22]. In short, lipase catalyzes the first step of the lipid by hydrolysis and produces free fatty acids, followed by the production of conjugated hydroperoxy fatty acids through lipoxygenase and finally, the formation of volatiles such as carbonyl compounds. In plants, 9- and 13-hydroperoxy-octadecadienoates are transformed into reactive intermediates by hydroperoxide dehydrases, hydroperoxide lyases, epoxide hydrolases, and hydroperoxide epoxygenase [21,23]. For example, the oxidation of linolenic acid by a lipoxygenase and a subsequent lyase cleavage reaction produces *trans*-2, *cis*-6-nonadienal in cucumbers, and *trans*-2-hexenal in fresh tomatoes. Moreover, these carbonyl compounds may further degrade into alcohol (e.g., *trans*-2, *cis*-6-nonadienol and *trans*-2-hexenol), which provides a stronger aroma than the precursor of carbonyls [16] (Figure 2). Apart from lipoxygenase, alcohol dehydrogenase, hydroperoxide lyase, and 3Z, 2E (3*cis*, 2*trans*) enal isomerase are also involved in the biosynthetic pathway. For instance, hydroperoxy fatty acids further break down into C6 or C9 volatiles such as 3-hexenal and 3,6-nonadienal, respectively, in the presence of hydroperoxide lyase [6].

In addition to the enzymatic and non-enzymatic pathways, there is a heat-mediated approach that also initiates lipid oxidation. The thermal oxidation of frying oil is the effect of the development of a complex form of the decomposition of fatty acids. The formation of cyclic and dimeric compounds leads to the thermolytic reaction in unsaturated fatty acids. Even saturated fatty acids become oxidized when heated at a high temperature (~150 °C) and produce *n*-alkanols, *n*-alkanes, lactones, 2-alkanones, 1-alkenes, and carboxylic acid [24].

Several analytical techniques have been used to identify and quantify lipid oxidation products. Generally, techniques that are used to measure the changes in primary oxidation products are changes in the fatty acid contents, peroxide value (PV) (e.g., iodometric titration and ferric-xylenol orange), and conjugated dienes/trienes, while the thiobarbituric acid reactive substances (TBARS) assay, *p*-anisidine value, TOTOX value (2 PV + *p*-anisidine), and volatile measurements using GC-MS are used to determine secondary oxidation products. Both primary and secondary oxidation products are recommended to be measured for better reliability regarding the state of oxidation.

## 4. Flavor Chemistry: Lipid Participation in Maillard Interaction

**Figure 3:**
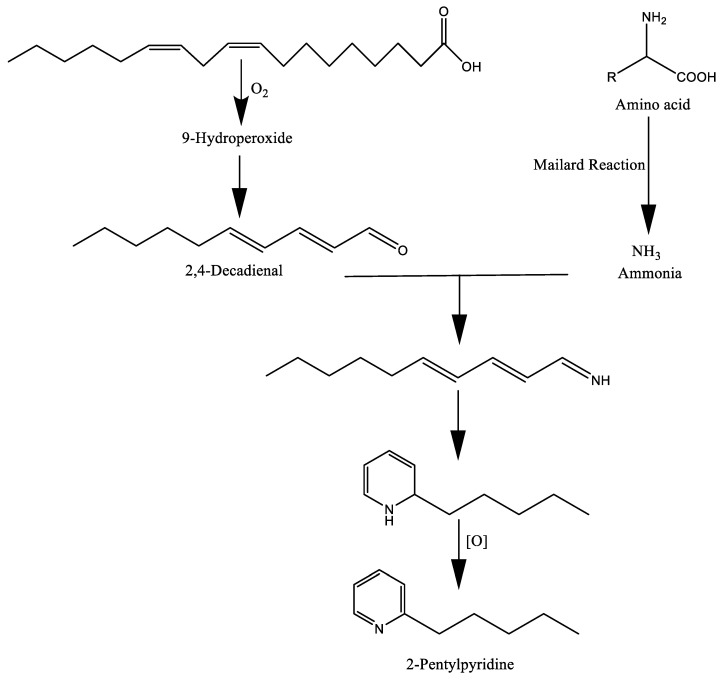
The formation of 2-pentylpyridine by lipid–Maillard interaction.

The Maillard reaction involves a series of complex chemical reactions between the carbonyls of reducing sugars and proteins, mainly primary or secondary amines, which is responsible for the browning of food and its distinctive flavor [25]. The Maillard reaction develops a wide range of chemical constituents belonging to oxazole, thiophene, furan, thiazole, pyrrole, pyrazine, and pyridine, among others. Generally, compounds such as peptides, amino acids, thiamine, carbohydrates, nucleotides, and lipids in food promote the development of flavor. Hence, the generation of aroma volatiles is basically related to the break down and oxidation of lipids, the Maillard reaction, and the degradation of vitamins, mainly thiamine [11]. For example, the Maillard reaction between ribose and cysteine produces sulfur-containing compounds including thiophan-3-thiol and furan-3-thiol, which are responsible for the desirable meaty aroma of cooked meat [9]. In particular, the products of these reactions can act as precursors and interact with other degradation constituents of food and produce a number of long-chain heterocyclic compounds during cooking. For instance, Henderson and Nawar [26] reported the formation of 2-pentylpyridine through the interaction of lipid (linoleic acid) and Maillard reaction by-products (Figure 3). Amadori products are produced during the first stages of the reaction via glycosylamine because of the interaction between the carbonyls of reducing sugars and primary amines of amino acids, peptides, or other components. The formation of Strecker aldehydes through the degradation of amino acids and lipid oxidation is another example of developing aroma compounds [27]. These compounds react with Maillard reaction-derived carbonyls and form intermediates, which further break down into flavor compounds. Apart from amino acids, phenolic compounds may promote the generation of Strecker aldehydes. For example, amino acids and quinones originating from phenolics could produce flavor precursor volatile aldehydes through Strecker degradation [28]. The same study also reported that amino acids (e.g., phenylalanine and methionine) were capable of interacting with phenolic compounds (e.g., chlorogenic acid, caffeic acid, epicatechin, and catechin) and developing Strecker aldehydes (e.g., phenylacetaldehyde and methional) in a ferricyanide-based model system.

Numerous sugar dehydration and degradation compounds such as dicarbonyl compounds, furanone and furfural derivatives, and hydroxy ketones are developed by the dehydration and rearrangement of the subsequent compounds [6,29]. However, the formation of Maillard reaction products depends on the moisture content, cooking temperature and time, pH, and the nature of the reactants involved. The reaction rate increases markedly with the temperature at low moisture levels, and the production of flavor compounds is linked with areas of the food dehydrated by heat. Moreover, the formation of Maillard reaction products is affected by the presence of a discrete non-polar environment and the reactants’ location. For example, the interaction between lipid oxidation and Maillard reaction products was examined in a model system of oil-in-water emulsion containing canola oil, water, glucose, phenylalanine, and a surfactant (Tween 20) [30]. It was found that the development of Maillard reaction products was 16 times greater in the emulsion system than in the aqueous solution.

Maillard reaction produces a myriad of components that are involved in flavor generation alone or with the lipid oxidation products. The interaction of these products produces new volatile compounds in some cases, or partially/wholly blocks other compounds [10]. For instance, aldehydes generated from lipid oxidation could participate in the initial and later stages of the Maillard reaction during cooking to form volatiles including pyrazines, thiophenes, pyridines, oxazoles, and thiazoles with alkyl side chains [11,31]. Several thiazoles and thiophenes such as 2-alkyl-3-thiazolines, 2-alkylthiazoles, and 2-alkylthiophenes have been reported in roasted meat. Particularly, carbonyls and sulfur compounds derived from ribose and cysteine are the major precursors to the flavor of meat. It has been reported that phospholipid oxidation produced 2,4-decadienal, which participated in the Maillard reaction and developed 2-alkyl heterocyclic products [9]. Usually, volatiles obtained from the combination lipid–Maillard reaction exhibit a higher odor threshold than those developed from its parent reaction, resulting in a weak odor intensity from the lipid–Maillard by-products. However, it may modify the aroma profiles obtained from this complex system and provide an indirect impact on the aroma compounds [10,11].

## 5. Effect of Processing on the Flavor Compounds of Meat and Eggs

Effect of various processing methods on major flavor components of different types of food is summarized in Table 1. 

Generally, salty, metallic, and bloody, with a bit of sweet aroma, are the common flavor of raw meats. Upon processing, mainly heating, meat and meat products produce a series of reactions including lipid oxidation, which are responsible for the development of specific meat flavors. During the thermal processing of meat, volatile compounds such as alcohols, ketones, aldehydes, furans, esters, hydrocarbons, carboxylic acids, pyrans, pyrazines, lactones, phenols, pyrroles, pyridines, thiazoles, thiazolines, oxazoles, thiophenes, and other nitrogen- or sulfur-containing compounds are formed [47]. Peptides, amino acids, nucleotides, organic acids, and other flavor enhancers are the most taste-active constituents of meats. Hundreds of volatile compounds have been characterized from various muscle foods, but no single component has so far been documented as being solely responsible for the aroma development, though hexanal has been found to be the predominant volatile in cooked meat. For example, Mottram [48] reported around 900 volatiles from cooked beef in which only a small number of this wide range of components contributed to the flavor profile. It has been reported that around 3% out of 10,000 identified volatiles contributed to the flavor development in foods [49]. During heat processing, lipids in the Maillard reaction produce species-specific flavors in meats. Generally, carbohydrates and free amino acids of different meats are similar, and thus, a similar flavor profile is expected upon cooking. Nevertheless, lipids in meat from different species are not always the same, especially intramuscular lipids, which modify the flavor profile upon heating. Particularly, the development of meaty aromas is mainly responsible for phospholipids present in the intramuscular lipids. Moreover, the impact of phospholipids is much higher than that of triacylglycerols (TAG) in terms of flavor generation due to the higher proportion of unsaturated fatty acids (e.g., arachidonic acid) in phospholipids [50,51]. Due to the presence of medium-chain branched fatty acids, the distinct strong flavor of lamb or mutton meat is observed. The adipose tissue of lamb meat contains 4-ethyl- and 4-methyloctanoic acids, which are responsible for the distinct flavor [6]. In contrast, chicken has a higher level of unsaturated fatty acids than red meat, resulting in the generation of more volatile aldehydes that are related to the distinct aroma of chicken. In particular, lipid-derived carbonyls, mainly aliphatic aldehydes, are responsible for the fatty flavors of roasted chicken meat. For example, 193 flavor compounds were identified from the roasted chicken; among them, 41 were lipid-derived aldehyde compounds [32]. They also reported that primary oxidation products of linoleic acid such as hexanal and 2,4-decadienal were the major flavor compounds in chicken meat. Apart from this, heat treatment also helps in lipid migration, which has both positive and negative effects on food quality. Xiang et al. [40] reported that lipid migration caused by heating promotes more lipid-specific flavor volatiles including 3-hydroxy-cyclohexanone, hexanal, D-limonene, 2-pentyl-furan, phenylacetaldehyde, and 2-ethyl-1-hexanol in the upper part of egg yolk.

Frozen storage also affects the flavor and sensory characteristics of meat due to the lipid oxidation catalyzed by iron and myoglobin. For instance, the effect of frozen storage on the flavor profile of marinated raw beef was investigated, and it was found that the concentration of flavor compounds including octanal, phenylacetaldehyde, 2-ethyl-1-hexanol, hexanal, 1-heptanol, and isoeugenol fluctuated along with the frozen storage [34]. Most of these compounds belong to alcohols and aldehydes, indicating that they were derived from lipid oxidation. This is because unsaturated fatty acids (e.g., linolenic, linoleic, and oleic acids) can undergo autoxidation or enzymatic oxidation and produce various hydroperoxides (e.g., 8-, 9-, 10-, 11- or 13-ROOH) and then volatiles (e.g., aldehydes) after homolysis. Similarly, the flavor of raw chicken meat was evaluated during frozen storage (0–8 weeks) [33]. It was found that the short-term frozen storage of raw meat improved its flavor profile. This could be due to the lipolysis, proteolysis, and oxidation degradation of flavor compounds in the long-term. Moreover, flavor components in cooked beef meatballs during frozen storage (0 to 90 days) were measured using various techniques, and 80 volatiles were identified in which 32, mainly 1-octene-3-ol, hexanal, 2-ethyl hexyl acetate, linalool, eugenol, diallyl disulfide, anisole, and α-pinene, were aroma-active [35]. Their sensory evaluation results demonstrated that the overall aroma profile started to decrease with the frozen storage.

Apart from this, the fermentation of meat improves its flavor and inhibits lipid oxidation. Chen et al. [36] suggested that fermentation of Harbin dry sausages lowered the number of volatiles (e.g., alcohols, aldehydes, hydrocarbons, and acids) generated from lipid autoxidation. Moreover, dry-aging is a common method for the development of distinct flavors due to microbial activity, dehydration, and lipid oxidation during storage. Particularly, dry-aging involves lipid oxidation and produces intermediates, which may interact with other flavor precursors such as peptides, amino acids, and sugars during heating and generate a dry-aged flavor [52]. On the other hand, the volatile profile of wet-cured cooked ham was compared with non-cured meat [37]. Carbonyls found in the cured meat were lower than the non-cured meat, and the major volatiles were terpenes (1,8-cineole, linalool, L–carvone, cinnamaldehyde menthol, and cinnamaldehyde), which mainly developed from seasonings, and sulfur components and 3-methylbutanoic acid originating from Strecker degradation. In contrast, aldehydes, ketones, and alcohols play a significant role in the flavor characteristic of dry-cured ham. Among the various volatiles, benzaldehyde, hexanal, 2-heptanone, limonene, hexanol, octanol, pentanol, 3-methylbutanal, 2-nonanone, butanol, and propanone were abundant in the dry-cured products [38,39].

### Factors Influencing the Flavor Formation of Meat

Several factors including the type of meat, the parameters of the production process, and components of meat are responsible for its flavor development. For example, the common volatile compounds of cooked beef are octanal, 2,4-decadienal, nonanal, methional, 2-furfurylthiol, 2-metyl-3-furanthiol, methanethiol, 3-mercapto-2-pentanone, and 4-hydroxy-2,5-dimethyl-3-(2*H*)-furanone. Interestingly, an almost similar flavor profile also occurs in cooked chicken and pork, but their concentrations differ among species. A high concentration of 2-methyl-3-furanthiol, 4-hydroxy-2,5-dimethyl-3-(2*H*)-furanone, and 2-furfurylthiol is associated with the meaty-caramel odor in cooked beef. In contrast, a lower concentration of 4-hydroxy-2,5-dimethyl-3-(2*H*)-furanone is enough to provide the odor of pork meat, and similarly, a lower level of carbonyls (e.g., hexanal and nonanal) is responsible for the greasy odor in pork than in beef meat [6,53].

Breed, sex, feed, nutrition, and age notably influence the quality and quantity of fats, which affect the overall flavor profile of meats. Fats and fatty acid composition are the principal contributors to the flavor generation in meat. The composition of fatty acids is mainly dependent on diet, and the flavor is produced upon melting fats. For instance, grass-fed beef is less susceptible to lipid oxidation than grain-fed beef, and this is because of the reduced content of flavonoids, vitamins A, C, and E, and carotenoids present in feeds [54]. Due to the differences in the digestive system, fatty acids, mainly polyunsaturated fatty acids, deposition is higher in beef or lamb. Thus, the meaty flavor is generated by the breakdown of these fatty acids upon degradation to aldehydes, ketones, and alcohols, among others. Apart from the type and content of fatty acids, protein content, temperature, time, water activity, reaction media, pH, aging, marbling, and cooking technique affect the flavor of meat [27]. Cooking methods modify the chemical composition of meat and promote lipid oxidation. Compared to other cooking methods, roasting results in increased oxidation and volatiles due to the longer time and high temperature experienced during cooking. Hence, the generation of flavor is increased through the Maillard reaction and lipid oxidation [55]. Similarly, grilling increases the content of pyrazines, a series of nitrogen-containing compounds responsible for the nutty and roasty flavor, which contribute up to 80% to the volatile compounds in grilled meat. Moreover, microwave cooking at a lower temperature and shorter time also causes increased lipid oxidation, which could be due to the interaction between microwave and meat fat. Frying can change the fatty acid composition due to added oil for frying, thus increasing the lipid oxidation [55,56,57].

## 6. Effect of Processing on the Flavor Compounds of Fish

**Figure 4:**
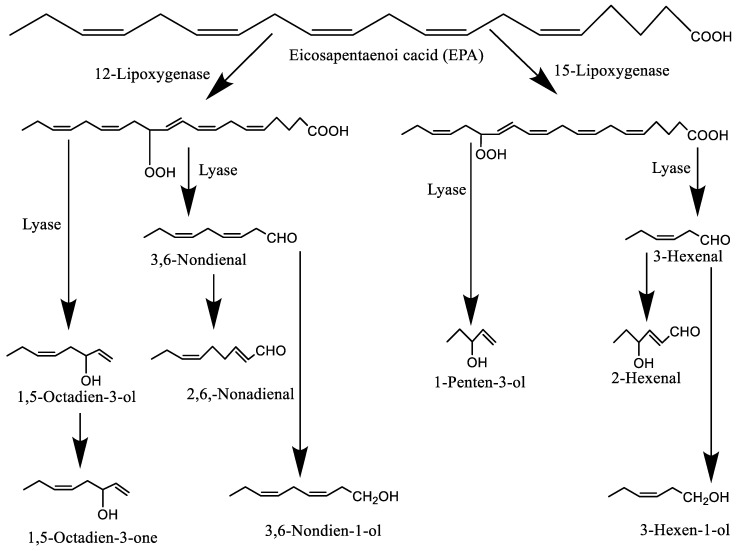
The enzymatic breakdown of eicosapentaenoic acid (EPA) (adapted from Shahidi [16]).

Both enzymatic and non-enzymatic oxidative reactions are involved in the development of fish aromas and flavors. The distinctive plant-green aroma of fresh fish is mainly produced from lipoxygenase-derived carbonyls and alcohols, which depends on the type and content of compounds present in different species. Lipoxygenase available in fish can generate alcohols and then further break down into carbonyls such as 1,5-octadien-3-one (Figure 4). Moreover, secondary oxidation products play the main role in the flavor development in certain species of fish [58]. The fresh fish aromas are linked mainly with alcohols (l-penten-3-ol, *cis*-3-hexen-l-ol, l-octen-3-ol, *trans*-2- octen-1-ol, l,5-octadien-3-ol, 2,5-octadien-l-ol, *trans*-2-nonen-l-ol, *cis*-6-nonen-l-ol, *cis*-3-nonen-l-ol, and 3,6-nonadien-l-ol) and carbonyls (*trans*-2-penten-l-al, hexan-l-al, *trans*-2-hexen-l-al, *trans*-octen-l-al, *trans*-2-nonen-l-al, l-octen-3-one, 2,3-octadien-l-one, and 1,5-octadien-3-one) [41]. Both desirable and non-desirable flavors are the result of lipid oxidation in marine-based food. In particular, secondary oxidation products including 2,4,7-decatrienals and other carbonyls of omega-3 fatty acids are responsible for the fishy off-flavor [9]. Apart from volatiles in seafood, non-volatiles including nucleotides, free amino acids, peptides, minerals, and sugars are also responsible for the development of taste and flavor [59]. Upon processing, mainly cooking, the flavor of fish changes dramatically, where lipid oxidation, Maillard reaction, and Strecker degradation play a leading role in generating meaty aromas. For example, during tuna canning, 2-methyl-3-furanthiol is produced upon the thermally mediated reaction of cysteine and ribose, which provides the meaty flavor. Some fish (e.g., conger, sardine, and pale chub) produce a distinct odor upon cooking and these are related to l-penten-3-ol, 2-phenylethanol, and dimethyl sulfide [41]. Moreover, carbonyls are the most important compounds in salted and dried fish. However, [60] reported that drying such as hot air, microwave, and microwave-vacuum drying removed a part of the fishy off-odor (e.g., 2-methylisoborneol and cyclic alcohols) and increased the grilled flavor of silver carp slices. However, the level of volatiles generated through lipid oxidation is lower in hot air-dried samples than those prepared by sun-drying [61]. On the other hand, Ke et al. [18] suggested that cold plasma-treated air enhanced the development of volatile flavor compounds in dry-cured fish originating from the oxidation of unsaturated fatty acids. The major volatiles were aldehydes (3-methylbutanal, octanal, 2-nonenal, n-hexanal, nonanal, 2,4-decadienal, and 2,4-nonadienal), alcohols (1-octene-3-ol), and ketones (1-octene-3- one). Among them, n-hexanal derived from the degradation of linoleic acid is responsible for the tallowy and green leafy notes to the fish flavor.

## 7. Effect of Processing on the Flavor Compounds of Edible Oils

Edible oils are mainly composed of TAG (~98%) and other minor components such as glycolipids, phospholipids, waxes, tocopherols, sterols, chlorophylls, and other phenolic compounds. These minor components are collectively referred to as unsaponifiable matters. During processing, many of these compounds are removed. In particular, chlorophylls in oils are responsible for the generation of odor-active aldehydes under fluorescent light [62,63]. However, the most common flavor components of fats and oils are generated from unsaturated fatty acids in TAG or polar lipids upon reacting with oxygen. For example, the major aldehydes produced upon oxidation of linolenic acid are propanal, 2-pentenal, 2-butenal, 2-hexenal, 3-hexenal, 2,4-heptadienal, 2-heptenal, 2,5 octadienal, 2,6-nonadienal, and 2,4,7-decatrienal, whereas pentanal, heptanal, hexanal, 2-octenal, octanal, 2-heptenal, 2-nonenal, 3-nonenal, 2,4-decadienal, 2-decenal, and 2,4-nonadienal are formed from the oxidation of linoleic acid and heptanal, octanal, decanal, 2-undecenal, decanal, and 2-decenal are developed from oleic acid [53]. Generally, the aromas of aldehydes are painty, metallic, green, beany, and rancid, and they are frequently related to the off-flavor. For instance, the main flavor compound responsible for deep-fat fried foods is 2,4-decadienal. This compound can further break down to *trans*-epoxy-*trans*-decenal, which is considered as one of the most effective odorants of soybean oil [64]. However, 2-pentylfuran and 3-*cis*-hexenal are mainly responsible for the beany or grassy flavor of soybean oil [65]. The cooking, mainly frying, of oils plays an important role in flavor development. For instance, Lee et al. [42] found that the frying of soybean and canola oils increased volatile concentration, and 2-heptenal, ethyl butyrate, and 2,4-pentanedione were the major volatiles in soybean oil, whereas ethyl butyrate and linalool were abundant in canola oil. Moreover, Xiao et al. [43] stated that the content of volatile aldehydes and alcohols was developed at 120 °C, whereas ketones and furans were produced at 150 °C, and acids were formed at 180 °C during the heating of soybean oil. The principal volatiles in most virgin or extra virgin olive oils (EVOO) are C5 and C6 aliphatic components such as 1-hexanol, hexanal, *trans*-2-hexenal, *trans*-2-hexen-1-ol, *cis*-3-hexen-1-ol, *cis*-2-penten-1-ol, and 3-methylbutanol [6]. However, the concentration of volatiles is species-specific and depends mainly on the fatty acid composition. Tian et al. [66] reported that the major volatile components of rapeseed, soybean, peanut, and sunflower oils were nearly the same including hexanal, *trans*-2-heptenal, nonanal, 2,4-decadienal, *trans*-2,4-nonadienal, *cis*-2-heptenal, 1-octene-3-ol, and 1-pentanol, but their concentration varied significantly.

## 8. Effect of Processing on the Flavor Compounds of Fruits and Vegetables

The major flavor precursors of fruits and vegetables are related to their fatty acids, which are mainly dependent on the degree of maturity, cultivar, geographic location, and processing methods. β-oxidation is the primary metabolic pathway for generating aroma compounds, whereas lipoxygenase also plays an important role in developing flavor compounds from fatty acids. For example, the biosynthesis of lactones is related to the flavor development of pineapple (δ-octalactone), coconut (γ-octalactone), peach and nectarine (γ-decalactone and γ-dodecalactone, respectively) via the β-oxidation pathway. In contrast, the degradation of linoleic and linolenic acids to aldehydes, alcohols, acids, and esters via the lipoxygenase pathway is responsible for the flavor development of fruits and vegetables [67,68]. For instance, lipoxygenase-derived volatiles including *cis*-3-hexenol, *cis*-3-hexenal, *trans*-2-hexenol, and *trans*-2-hexenal, which are related to the fresh green flavor of tomatoes [69]. However, various processing methods have a significant effect on the flavor profile of fruits and vegetables. Shan et al. [44] reported that the bagging treatment enhanced the flavor quality of cucumbers, which were related to *trans*-2, *cis*-6-nonadienal, and *trans*-2-nonenal. Similarly, Lomelí-Martín [70] suggested that high hydrostatic pressure increased the content of aldehydes and ketones and decreased that of alcohols in the fruits and vegetables. This could be due to the enzymatic activities and chemical reactions upon high-pressure treatment. For example, hexanal is associated with the smell of foliage and grass, which could increase upon high-pressure treatment due to the oxidation of free fatty acids [71]. Furthermore, autoxidation and the Maillard reaction are the major chemical reactions responsible for the flavor development of fruits and vegetables during drying. Zhang et al. [45] found that drying methods (e.g., hot-air drying, freeze-drying, and natural drying) changed the contents of volatiles (e.g., 1-octen-3-one, 1-octen-3-ol, and 3-octanone) in mushroom and resulted in a weaker mushroom flavor. Likewise, the volatiles (e.g., 2-methylbutanal, 3-methylbutanal, n-hexanal, 6-methyl-5-hepten-2-ol, 3-methyl-1-butanol, and 6-methyl-5-hepten-2-one) of tomatoes were higher in the oven-dried than the sun-dried samples, indicating that volatiles were mainly formed by oxidation during the thermal treatment upon oven drying [46]. 

## 9. Volatile Measurements

Various techniques can be used to assess volatiles such as aldehydes, ketones, furans, and alcohols. Headspace analysis is a very common method of determining volatiles. Generally, gas chromatography coupled with a flame ionizing detector (FID) or mass spectrometry (GC-MS) and/or an olfactory port is used to analyze volatiles [10]. Steam distillation-extraction (SDE), solid-phase microextraction (SPME), and dynamic headspace are very common methods to extract volatiles. Moreover, it has been reported that the combination of two or more methods (e.g., gas chromatography-olfactometry, GC-O) results in the better separation of volatiles. In particular, GC-O techniques including combined hedonic response measurement, aroma extraction dilution analysis (AEDA), aroma extract concentration analysis (AECA), surface of nasal impact frequency (SNIF), and finger span cross modality (FSCM) are useful to determine the intensity of aroma (odor) and the characteristics of volatiles [9,55]. Among them, AEDA is the most common technique used to analyze volatiles due to its simplicity. Furthermore, multidimensional gas chromatography (MDGC) coupled with MS and olfactometry detection has recently been used to investigate the flavor profile [72]. Apart from these, the electronic nose (E-nose) has gained in popularity in determining the volatile profiles. For example, Xu et al. [73] suggested that the E-nose has a huge potential for the rapid inspection of volatiles (e.g., aldehydes, alcohols, 2-acetyl-1-pyrroline, and heterocycles) in rice aging.

## 10. Conclusions

The development of volatile components in various foods mainly depends on their constituent compounds and how they process. The generation of flavor volatiles is primarily linked to lipid degradation, the Maillard reaction, and interactions between the Maillard reaction and lipid oxidation compounds, among others. Processing such as slow cooking is mostly responsible for lipid oxidation, while fast cooking is associated with the generation of Maillard reaction products. Hence, the development of off-flavor can be controlled, and a desirable flavor can be produced by understanding the detailed mechanisms and inclusion of biologically active compounds.

## Figures and Tables

**Table 1:** Important flavor components of selected food.

Processing Method	Type of Food	Volatile Compounds	References
Roasting	Chicken	Butanal, pentanal, hexanal, octanal, nonanal, hexadecanal, octadecanal, 2-metbylpyrazine, 2,3-dimetbylpyrazine, pyridine, 2-methylpyridine, *N*-metbylpyrrole, and 2-methylthiazole	[32]
Cooking	Chicken	Hexanal, heptanal, octanal, nonanal, hexadecanal, *trans*-2-pentenal, *trans*-2-heptenal, *trans*-2-octenal, *trans*-2-decenal, *trans*-2-undecenaI, *trans,cis*-2,4-decadienal, and *trans,trans*-2,4-decadienal	[32]
Frozen storage	Chicken	1-Octene-3-ol, hexanal, 2-ethyl hexyl acetate, linalool, eugenol, diallyl disulfide, anisole, and α-pinene	[33]
Cooking	Beef	Pentanal, hexanal, heptanal, nonanal, 12-methyltridecanal, nona-2-*trans*-enal, decan-2-one, 1-cctene-3-ol, pyrazines, 2-methyl-3-furan, 2-pentyl furan	[1,6]
Frozen storage	Beef	Octanal, phenylacetaldehyde, 2-ethyl-1-hexanol, hexanal, 1-heptanol, and isoeugenol	[34]
Frozen storage	Meatballs (beef)	1-Octene-3-ol, hexanal, 2-ethyl hexyl acetate, linalool, eugenol, diallyl disulfide, anisole, and α-pinene	[35]
Fermentation	Sausages (pork)	Hexanal, heptanal, decanal, nonanal, *trans*-cinnamaldehyde, 2-heptanone, 3-hydroxy-2-butanone, linalool, terpinen-4-ol, and ethyl acetate	[36]
Modified-atmosphere packaging	Cooked ham	1,8-Cineole, linalool, L–carvone, cinnamaldehyde menthol, and cinnamaldehyde	[37]
Curing	Ham	Benzaldehyde, hexanal, 2-heptanone, limonene, hexanol, octanol, pentanol, 3-methylbutanal, 2-nonanone, butanol, and propanone	[38,39]
Boiling	Egg yolk	3-Hydroxy-cyclohexanone, hexanal, D-limonene, 2-pentyl-furan, phenylacetaldehyde, and 2-ethyl-1-hexanol	[40]
Cooking/canning	Tuna, conger, sardine, and pale chub	2-Methyl-3-furanthiol, l-penten-3-ol, 2-phenylethanol, and dimethyl sulfide	[41]
Cold plasma treatment	Cured black carp	3-Methylbutanal, octanal, 2-nonenal, n-hexanal, nonanal, 2,4-decadienal, 2,4-nonadienal,1-octene-3-ol, ketone 1-octene-3-one	[18]
Frying	Soybean and canola oils	2-Heptenal, ethyl butyrate, 2,4-pentanedione, acetyl pyrazine, 1-octanoland, 3-methylbutanal, pyridine, and linalool	[42]
Heating	Soybean oil	Butanal, pentanal, hexanal, heptanal, octanal, nonanal, decanal, undecanal, dodecanal, *trans*-2-butenal, *trans*-2-pentenal, *trans*-2-hexenal, *cis*-4-heptenal, *cis*-2-heptenal, *trans*-2-octenal, *cis*-2-nonenal, *cis*-2-decenal, *cis*-2-decenal, 2-undecenal, *trans*-2-hepten-1-o, 2-butanone, and 2-pentylfuran	[43]
Bagging	Cucumber	*trans*-2, *cis*-6-Nonadienal, *trans*-2-nonenal, nonanal, *n*-hexanal, *trans*-2-hexenal, propanal, and *cis*-2-heptenal	[44]
Drying	Mushroom	1-Octen-3-one, 3-octanone, 1-octen-3-ol, 3-octanol, 2-octen-1-ol, 1-octanol, benzaldehyde, benzeneacetaldehyde, and decanal	[45]
Drying	Tomato	2-Methylbutanal, 3-methylbutanal, *n*-hexanal, 6-methyl-5-hepten-2-ol, 3-methyl-1-butanol, and 6-methyl-5-hepten-2-one	[46]

## Data Availability

Not applicable.
